# Seroprevalence of Tick-Borne Encephalitis Virus in Latvia Using Standard and Subtype-Specific NS1-Based ELISA Assays

**DOI:** 10.3390/pathogens14111115

**Published:** 2025-11-02

**Authors:** Zane Freimane, Gerhard Dobler, Guntis Karelis, Lidia Chitimia-Dobler, Oksana Savicka, Dace Zavadska

**Affiliations:** 1 Department of Paediatrics, Rīga Stradinš University, 1007 Riga, Latvia; 2 Children’s Clinical University Hospital, 1004 Riga, Latvia; 3 Bundeswehr Institute of Microbiology, National TBEV Consultant Laboratory, 80937 Munich, Germany; 4 Department of Tropical Medicine and Infectious Diseases, Ludwig Maximilians University, 80802 Munich, Germany; 5 Department of Parasitology, University of Hohenheim, 70599 Stuttgart, Germany; 6 Department of Neurology and Neurosurgery, Riga East University Hospital, 1079 Riga, Latvia; 7 Department of Infectology, Rīga Stradinš University, 1007 Riga, Latvia; 8 Research Professor (Tenured Professor) Group in Neuroimmunology, Department of Biology and Microbiology, Faculty of Medicine, 1007 Riga, Latvia; 9 Fraunhofer Institute for Translational Medicine and Pharmacology ITMP, Immunology, Infection and Pandemic Research IIP, Tuerkenstraße 89, 80799 Munich, Germany; 10 National Microbiology Reference Laboratory, Riga East University Hospital, 1006 Riga, Latvia

**Keywords:** Latvia, TBEV antibodies, seroprevalence, NS1 ELISA, orthoflavivirus

## Abstract

*Background:* Latvia is one of the most endemic tick-borne encephalitis (TBE) countries in Europe. However, assessing TBE seroprevalence and true infection rates has been challenging. Current diagnostics cannot reliably distinguish between immune responses induced by natural infection from those induced by vaccination, especially in TBE endemic countries with recommended immunisation programmes. A recently developed ELISA targeting antibodies against TBEV non-structural protein 1 (NS1) offers improved specificity for natural infection and can differentiate among three clinically relevant TBEV subtypes. *Methods:* We conducted a cross-sectional TBEV seroprevalence study in the Latvian population during 2019–2022, consisting of two pools: 1020 residents living in different regions of Latvia and 200 random Latvian Biobank blood samples. We used the standard anti-TBEV IgG ELISA (VIDITEST, VIDIA, Czech Republic) for the screening and detection of TBEV (whole virus) IgG antibodies and the newly established research-use anti-TBEV NS1 IgG ELISA for the detection of subtype-specific TBEV NS1 IgG antibodies against three virus subtypes: European, Siberian and Far Eastern. *Results:* The total TBEV seroprevalence among 1020 residents was 39.7%, representing all age cohorts of the population from all regions of Latvia. In total, 33.4% of the enrolled population were vaccinated against TBE with at least one dose of the TBE vaccine. Among the unvaccinated population, 16.3% had positive TBEV-specific IgG antibodies by standard ELISA. On the contrary, NS1-specific antibodies, indicating past natural infection, were detected in only 4.3% of the overall study population. Subtype differentiation revealed infections from all three major TBEV subtypes present in Latvia. *Conclusions:* In conclusion, this population-based study highlights a high risk of TBE in Latvia, with substantial seroprevalence even among unvaccinated individuals. The NS1-based ELISA enhances the accuracy of TBE surveillance and offers important clinical utility by facilitating more reliable diagnosis and case classification, regardless of vaccination status.

## 1. Introduction

Tick-borne encephalitis (TBE) is a viral infectious disease of the central nervous system (CNS) caused by the tick-borne encephalitis virus (TBEV) [1]. TBEV is predominantly transmitted to humans by the bite of an infected *Ixodes* tick and, less commonly, by the consumption of unpasteurised dairy products [1,2]. TBE is a significant public health concern in many parts of the world, particularly in Europe [1,2,3]. In 2022, 20 European Union/European Economic Area (EU/EEA) countries reported 3650 cases of tick-borne encephalitis (TBE), 3516 (96.3%) of which were confirmed [4]. Latvia is a northern European country with a historically high incidence of TBE. Between 2018 and 2020, a population-based study in Latvia reported a high average incidence of TBE—10.3 TBE cases per 100,000 population; moreover, the incidence increased to 31.7/100,000 TBEV-infected cases in the non-vaccinated population of Latvia [5]. In 2022, Latvia reported the third-highest TBE incidence to the European Centre for Disease Prevention and Control (ECDC), after Lithuania and the Czech Republic [4].

In endemic countries, TBE continues to be one of the most frequent causes of viral meningitis/encephalitis. Although TBEV infections may be asymptomatic, most infections result in symptoms of CNS inflammation ranging from mild meningitis to severe encephalitis, usually requiring hospitalisation, and can result in long-term neurological sequelae and death. There is no specific treatment; however, highly effective vaccines against TBEV are available in Europe (FSME Immun, Pfizer; Encepur, Bavarian Nordic). Vaccination is recommended for people living in or travelling to endemic regions [6]. Although vaccination coverage in Latvia has been reported as up to 62% of the population being vaccinated with at least one TBE vaccine dose, the annual incidence of the disease remains high [7].

Despite its importance, the burden of TBE remains poorly understood in many regions, particularly in the population infected with TBEV. Seroprevalence studies and true infection rates have been challenging to access in TBE endemic countries with recommended immunisation programmes, as current diagnostic methods are not designed to distinguish immune responses induced by natural TBEV infection from those induced by vaccination. Enzyme-linked immunosorbent assay (ELISA) is considered the standard method for the serological diagnosis of TBE. However, the currently available ELISAs have the fundamental disadvantage of cross-reactivity with other flavivirus infections or vaccinations. This is largely due to the use of shared antigens—typically the TBEV structural envelope E protein, which is also present in inactivated vaccine formulations. Consequently, antibody responses induced by vaccination and those by natural infection are serologically indistinguishable. In contrast, the non-structural 1 (NS1) protein is expressed exclusively during active viral replication and is absent from inactivated vaccines. Therefore, antibodies against NS1 are produced only following natural TBEV infection, providing a reliable serological marker to distinguish natural infection from vaccine-induced seropositivity [8].

Recently, a new approach has been introduced to detect antibodies against the TBEV non-structural protein 1 applied to the in-house ELISA [9] and suspension multiplex immunoassay [8,10]. Anti-TBEV NS1 IgG ELISA (hereinafter denoted as NS1 IgG ELISA) is a rapid diagnostic method, useful for the routine diagnostic laboratory to detect immune response after exclusive wild-type natural TBEV infection, with the additional convenience of differentiating between three clinically relevant TBEV subtypes: European, TBEV-EU; Siberian, TBEV-Sib; and Far Eastern, TBEV-FE [11]. It is suggested that all three primary TBEV subtypes are present in Latvia, as the European and Siberian subtypes have been identified from patients with TBE, and the Far Eastern subtype has been isolated from ticks in Latvia [12,13]. It is assumed that the different subtype infections exhibit different clinical courses and outcomes; therefore, serological differentiation is important.

This study aimed to determine the seroprevalence of TBEV in Latvia, in both vaccinated and unvaccinated populations, using two different serological methods: the routinely used standard ELISA for the detection of TBEV (whole virus)-specific IgG antibodies and the newly established NS1 IgG ELISA for the detection of subtype-specific TBEV NS1 IgG antibodies. Our objective was to provide valuable information on the immunological status of the population against TBEV, including antibody frequency and distribution. We also aimed to describe the epidemiology and subtype distribution, as well as the proportion of asymptomatic or undiagnosed infections. These findings are intended to support public health strategies for disease prevention and control.

## 2. Method

### 2.1. Study Design and Participants

We conducted a cross-sectional TBE seroprevalence study in the Latvian population, consisting of two pools: 1020 residents living in different regions of Latvia and 200 random Latvian Biobank samples.

### 2.2. Residents from Five Different Regions of Latvia

The study included 1020 residents from five different regions of Latvia, covering the whole country—Kurzeme, Zemgale, Riga/Central region, Vidzeme, and Latgale. Two hundred participants were randomly enrolled from each region of Latvia, according to population age distribution, reported by the Central Statistical Bureau of Latvia in 2020. Each regional group represented approximately equal sample sizes (20.8% children “1–17 years old”, 24.5% adults “18–39 years old”, 34.3% adults “40–64 years old” and 20.5% adults “65+ years old”). Enrolment settings included family practices and hospitals. Enrolment was conducted between 10 September 2019 and 30 May 2022. Prior to enrolment, informed consent was obtained for all participants, and legal guardian consent was obtained for all children aged 1 to 17 years old, according to National Legislation.

### 2.3. Biobank Samples

The study included 200 anonymous random samples from the Latvian Biobank. Samples were frozen anonymously, and informed consent was not required. TBE vaccination status and medical history were unknown for the Biobank samples.

### 2.4. Data Collection and Procedures

After receiving informed consent from a participant (Group: Residents of Latvia), study staff collected information on age, gender, the region of residence, risk factors, tick bite history or consumption of unpasteurised dairy products, TBE vaccination history, and previous TBE infection history. In previously vaccinated individuals, vaccination status was verified by the vaccination passport, including vaccination dates. We utilised the anti-TBE Virus IgG ELISA (VIDITEST, VIDIA, Czech Republic)*,* hereinafter denoted as the standard ELISA, for the screening and detection of TBEV (whole virus) IgG antibodies, and the newly established, research-use anti-TBEV NS1 IgG ELISA (hereinafter denoted as NS1 IgG ELISA), developed in-house for the detection of subtype-specific TBEV NS1 IgG antibodies against three virus subtypes (TBEV-EU, TBEV-Sib, and TBEV-FE). Serological testing was performed at the National Microbiology Reference Laboratory in Riga, Latvia, and at the Bundeswehr Institute of Microbiology, National TBEV Consultant Laboratory, in Munich, Germany, where the sample results were independently confirmed.

### 2.5. TBEV Subtype Differentiation

We have previously described the development and validation of the NS1 IgG ELISA for the differentiation of three TBEV subtypes (European, Siberian, and Far Eastern) [11]. Recombinant NS1 proteins representing these subtypes were produced in HEK-293 human cell lines (The Native Antigen Company, Kidlington, UK), purified to preserve their native hexameric conformation, and used for ELISA coating as described earlier. For each serum sample, duplicate wells were tested in parallel against all three subtype antigens. Average optical density (OD) values were compared, and the subtype with the highest OD was considered the most likely causative subtype.

Positive and negative controls, consisting of pooled sera with known anti-TBEV IgG antibody levels, were included on each plate. Cut-off values were calculated as previously established [11]: the negative threshold was defined as the mean OD of negative sera +1 standard deviation (SD), and the positive threshold as the mean OD +3 SD. This approach has been shown to provide a sensitivity of up to 100% for TBEV-Eu and 96% for TBEV-Sib/FE differentiation, with an overall specificity of 99–100% when tested against sera from vaccinated individuals or those infected with other flaviviruses.

### 2.6. Statistical Analysis

Statistical analysis was performed in two groups: residents of Latvia in different regions (Group 1) and Biobank samples (Group 2). In Group 1, the prevalence of anti-TBEV IgG antibodies was calculated according to questionnaire data in the following groups: vaccinated and non-vaccinated individuals, age groups (<18, 18–39, 40–64, and 65+ years old) and different regions of endemicity. In Group 2, the prevalence of anti-TBEV IgG antibodies was calculated by the total number of samples due to a lack of detailed information, and vaccination status.

Statistical analyses were performed using chi-square or Fisher’s exact tests as appropriate, and *p*-values < 0.05 were considered significant. Confidence intervals (95% CI) were calculated for major comparisons. Statistical analyses were conducted with IBM SPSS Statistics version 22 and GraphPad Prism version 6 for Windows (GraphPad Software, San Diego, CA, USA).

### 2.7. Ethics Statement

This research was carried out in line with “The Code of Ethics of the World Medical Association (Declaration of Helsinki)” and according to good clinical practice guidelines. In accordance with local legislation, the study was approved by the Rīga Stradinš University Ethics Committee (No. 6-1/03/19; 26 March 2020). Written informed consent was obtained prior to enrolment. Biobank samples only included anonymous sera for research purposes; therefore, informed consent was not required.

## 3. Results

### 3.1. Group 1: Residents in Different Regions of Latvia

#### 3.1.1. Characteristics and Demographic Data

A total of 1020 participants were enrolled in the study during 2019–2022 from five different regions of Latvia—Kurzeme (*n* = 203), Zemgale (*n* = 205), Riga region (*n* = 202), Vidzeme (*n* = 203) and Latgale (*n* = 207). Based on the population data presenting median age cohorts in 2019–2022, the study included 18.2% children (*n* = 186) “1–17 years old”, 26.5% *(n* = 270) adults “18–39 years old”, 34.3% (*n* = 350) adults “40–64 years old” and 21.0% (*n* = 214) adults “65+ years old”. Of them, 59.6% (*n* = 608) were female and 40.4% (*n* = 412) were male (Table 1).

#### 3.1.2. TBE Vaccination Status

TBE vaccination status was known for 1019/1020 cases. It was not possible to ascertain the vaccination status against TBEV for one participant. Vaccination data revealed that 34.9% (*n* = 356) of participants had received at least one prior TBE vaccine dose. Of those vaccinated against TBEV, 37.1% (132/356) were fully vaccinated and received at least three doses according to the schedule. In total, 65% (663 residents) were not vaccinated against TBE.

#### 3.1.3. Risk Factors and Epidemiological Link

Information about occupational risk factors was available for 993/1020 participants; of them, 150 (15.1%) had occupational risk factors, most commonly farmer (*n* = 86), forest worker (*n* = 43), and hunter (*n* = 20). Occupational risk factors were seen more often in the non-vaccinated group, 56% (*n* = 84), versus the vaccinated group, 44% (*n* = 66). Information on leisure activity risk factors was available for 993/1020 participants. In total, 83.8% (*n* = 832) of participants had reported at least one leisure activity risk factor, such as walking/hiking (*n* = 724, 87%), berry/flower/mushroom picking (*n* = 583, 70.1%), outdoor sports (*n* = 295, 35.5%), and camping (*n* = 298, 35.8%). Tick bite history data were available for 980/1020 participants; among them, a tick bite was noticed in 425/980 (43.4%) participants, with 26 of those individuals reporting the bite within the last 30 days. Five patients reported previous TBE disease history.

#### 3.1.4. TBEV Seroprevalence by Standard ELISA and Subtype-Specific NS1 IgG ELISA

In Group 1, healthy residents of Latvia, including both vaccinated and non-vaccinated individuals, TBEV-specific IgG antibodies by the standard ELISA were detected as positive in 39.7% (*n* = 405) participants, borderline in 1.8% (*n* = 18), and negative in 58.5% (*n* = 597) participants.

The presence of three distinct (EU, Sib, FE) TBEV subtype-specific NS1 IgG antibodies was tested in 423 participants with borderline/positive TBEV-specific IgG antibodies by the standard ELISA. The TBEV subtype could be differentiated in 44 cases, or 4.3% (44/1020) of Group 1 overall, where the OD measurement of one subtype was higher than the ODs of the two other subtypes. Specifically, the TBEV-EU subtype was detected in 36/44 (81.8%) residents, TBEV-Sib in 7/44 (15.9%) residents, and TBEV-FE in 1 resident. This was an 87-year-old male currently living in the coastal region of Kurzeme; he had received four TBE vaccine doses starting from 2007 and reported no TBE history.

#### 3.1.5. TBEV Seroprevalence Among Five Different Regions of Latvia

**Figure 1:**
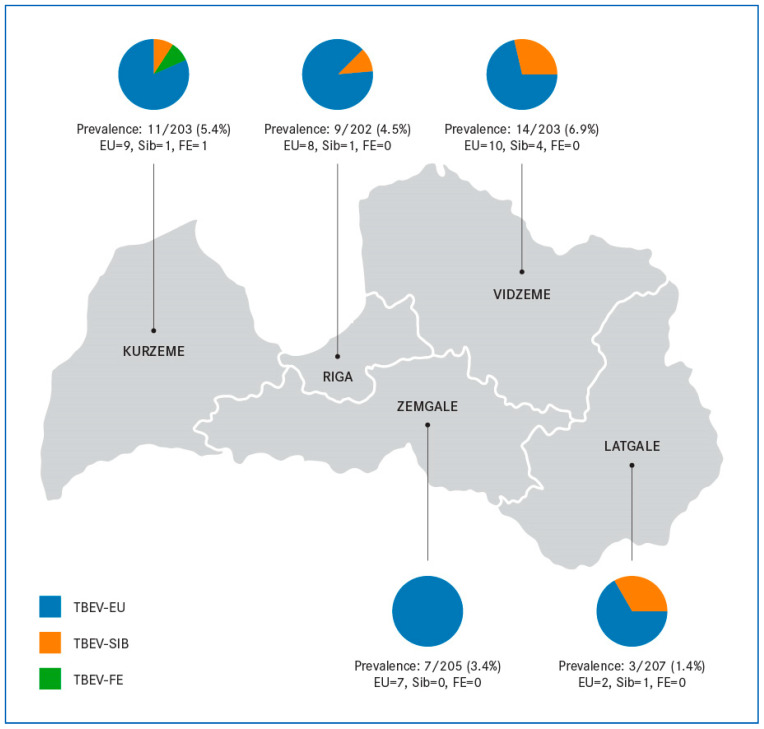
Tick-borne encephalitis virus (TBEV) NS1 IgG seroprevalence and subtype distribution across five regions of Latvia. Each region is outlined by borders of the Latvian administrative map. Pie charts illustrate the proportion of European (TBEV-EU, blue), Siberian (TBEV-Sib, orange), and Far Eastern (TBEV-FE, green) subtypes detected among NS1-positive individuals in that region.

The distribution of NS1-positive individuals varied across Latvia’s five regions (Figure 1). The highest number was observed in Vidzeme 14/203 (6.9%), Kurzeme 11/203 (5.4%), Riga/Central 9/202 (4.5%), Zemgale 7/205 (3.4%), and Latgale 3/207 (1.5%). However, the chi-square test results in a *p*-value of 0.087 (95% CI: 1.5–6.9%), indicating that the differences are not statistically significant.

Subtype differentiation showed that the TBEV-EU predominated in all regions, accounting for 81.8% of TBE cases in Kurzeme (9/11), 66.7% in Latgale (2/3), 88.9% in Riga (8/9), 71.4% in Vidzeme (10/14), and 100% in Zemgale (7/7). Seven patients with TBEV-Sib infection were detected throughout the whole country, with the highest numbers in the northeast region of Vidzeme (4/7 cases). The TBEV-Sib was detected in 1 case each from Kurzeme, Riga and Latgale, representing the minority subtype (ranging from 9.1% to 33.3% within regions). A single TBEV-FE case was identified in Kurzeme. Overall, these results confirm that TBEV-EU is the dominant subtype across Latvia, while TBEV-Sib circulates at lower frequencies, particularly in Vidzeme and Latgale, the eastern part of Latvia.

#### 3.1.6. TBEV Seroprevalence Among Non-Vaccinated Residents of Latvia

Among non-vaccinated individuals, the standard ELISA demonstrated TBEV seroprevalence of 16.3% (95% CI: 13.5–19.1%, *n* = 108), with the highest prevalence among the 18–39-year-old age group (22.0%, 95% CI: 15.5–28.5%, Chi-square test, *p* = 0.005). Latvian regions with the highest seroprevalence in non-vaccinated residents were Zemgale, 25.6% (31/189 cases), and the Riga/Central region, 19.7% (27/137 cases).

However, the NS1 IgG ELISA showed lower overall seroprevalence among non-vaccinated participants—a total of 2.9% (95% CI: 1.6–4.2%), with the highest prevalence among the “40–64” age group—4.5% (*p* = 0.515); differences across adult age groups were not statistically significant (Table 2).

#### 3.1.7. Seroprevalence Among Vaccinated Residents of Latvia

Among vaccinated individuals, the standard ELISA demonstrated seroprevalence of 83.4% (297/356; 95% CI: 79.2–86.9%), among children 84.7% (61/72; 95% CI: 74.7–91.2%), and adults 83.1% (236/284; 95% CI: 78.3–87.0%), with the highest prevalence observed in the 18–39-year age group (90.2%, 92/102; 95% CI: 82.9–94.6%). The age of participants ranged from 1 to 78 years (median 35 years). The difference in seroprevalence between age groups was statistically significant (chi-square test, *p* = 0.027).

The NS1 IgG ELISA showed an overall seroprevalence among vaccinated participants of 7.0% (95% CI: 4.8–10.2%; 25/356 cases), with no statistically significant difference between regions (Fisher’s exact test, *p* > 0.05). Participants originated from five Latvian regions, with the highest number from Vidzeme (*n* = 12), followed by Riga (*n* = 5), Kurzeme (*n* = 3), Zemgale (*n* = 3), and Latgale (*n* = 2). The identified TBEV subtypes were predominantly European (*n* = 18), followed by Siberian (*n* = 6) and Far Eastern (*n* = 1).

### 3.2. Group 2: Biobank Samples

A total of 200 Latvian Biobank samples were also included in the study. Vaccination status was unknown for all samples in this group. TBEV seroprevalence by the standard ELISA was 32.5% (95% CI: 26.2–38.8%; *n* = 65). A borderline result was detected in 2 samples (1.0%) and a negative result in 133 (66.5%).

The TBEV subtype could be differentiated in 12 of the 200 cases (6.0%), where the OD measurement of one subtype was higher than the ODs of the two other subtypes. Specifically, the TBEV-EU subtype was detected in 10/12 (83.3%) residents, and 2 residents indicated a previous TBEV-Sib infection.

### 3.3. Overall Results of Seroprevalence and Subtype Differentiation in Study Groups

The overall seroprevalence of TBEV-specific IgG antibodies, as determined by the standard ELISA, was 39.7% (95% CI: 36.7–42.7%) in healthy Latvian residents (Group 1) and 32.5% (95% CI: 26.2–38.8%) in Biobank samples (Group 2), with no statistically significant difference between groups (Chi-square test, *p* > 0.05). Among non-vaccinated individuals, seroprevalence was 16.3% (95% CI: 13.5–19.1%, *n* = 108), with the highest prevalence among the 18–39-year-old age group (22.0%, 95% CI: 15.5–28.5%).

The NS1 IgG ELISA detected subtype-specific antibodies in 4.3% (95% CI: 3.0–5.6%) of Group 1 participants and 6.0% (95% CI: 2.8–9.2%) of Group 2 participants, with no statistically significant difference between groups (Fisher’s exact test, *p* = 0.29). In NS1-positive individuals, subtype differentiation revealed prior infection with the TBEV-EU subtype in 81.8% (Group 1, Latvian residents in different regions) and 83.3% (Group 2, Biobank samples). TBEV-Sib accounted for 15.9% and 16.7% of cases in the two groups, respectively. A single TBEV-FE case was detected in Group 1. Overall, the seroprevalence measured by the NS1 IgG ELISA was significantly lower than that detected by the standard ELISA in both groups (*p* < 0.001), indicating a lower proportion of participants with evidence of past natural infection.

## 4. Discussion

Tick-borne encephalitis virus (TBEV) poses a significant public health concern in endemic areas of Europe and Asia. The detection of specific antibodies, particularly against the non-structural protein 1 (NS1) of TBEV, has become a valuable tool in distinguishing natural infection from vaccination-induced immunity [8,9,10]. This study investigates the seroprevalence of TBEV in both vaccinated and non-vaccinated individuals in Latvia using the standard TBEV IgG ELISA (VIDITEST, VIDIA Czech Republic) for the screening and detection of TBEV (whole virus) IgG antibodies and the newly established in-house NS1 IgG ELISA for the detection of subtype-specific TBEV NS1 IgG antibodies against three virus subtypes (European, Siberian and Far Eastern) [11]. A study representative of the population was designed to include residents (N = 1020) representing population age cohorts from all regions of Latvia. The study also included 200 anonymous Latvian Biobank samples.

Our findings indicate a notably high overall TBEV seroprevalence of 39.7% using the standard ELISA, representing the combined antibody responses resulting from both prior vaccination and natural infection. Using both the standard ELISA and the novel subtype-specific NS1 IgG ELISA, we were able to differentiate between vaccine-induced immunity and antibodies acquired through natural infection, offering a more nuanced understanding of TBEV exposure dynamics in the Latvian population. Further, the NS1 IgG ELISA revealed that overall NS1-positive seroprevalence in the study population was 4.3%. Notably, only 0.5%, or five study participants, reported prior, clinically confirmed TBE, underscoring the likely high proportion of asymptomatic or mild infections that remain unrecognised.

Among unvaccinated individuals, 16.3% tested positive for TBEV-specific IgG antibodies using the standard ELISA, suggesting significant prior exposure to TBEV, which was particularly notable among adults aged 18–39 years old (22.0%). However, when assessed with the more specific NS1 IgG ELISA, the seroprevalence attributable to natural infection was lower (2.9%). This discrepancy may reflect limitations in historical vaccination records, undiagnosed prior vaccinations or possible sensitivity and specificity limitations of the test. The lack of a comprehensive national electronic TBE vaccination registry for all age groups complicated the validation of self-reported vaccination history in non-vaccinated individuals, raising the possibility of recall bias among participants. These factors may collectively explain the observed data discrepancies.

An additional consideration for interpreting the observed discrepancies between standard ELISA and NS1 ELISA results is the role of virus neutralisation tests (NTs), which remain the gold standard for confirming TBEV infection [14]. NTs provide higher specificity by directly assessing functional virus-neutralising antibodies [15]. However, they have notable limitations: NTs may still show cross-reactivity with other flaviviruses circulating in Europe (e.g., West Nile virus), and importantly, they cannot differentiate between vaccine-induced and infection-induced antibodies. In our study, NTs were not performed because this methodology is currently not available in Latvia. Nevertheless, future investigations could incorporate confirmatory NTs in representative subsets of samples to better validate NS1-based assays and their diagnostic accuracy.

The application of the NS1 IgG ELISA in this study allowed for a more accurate assessment of natural TBE infection rates among the vaccinated population. In this study, 34.9% reported documented prior vaccination against TBEV. Vaccination coverage in this study is lower than the national estimate of 62% previously reported for 2020 [7]. This discrepancy likely reflects enrolment bias, as only individuals with verifiable vaccination records were included. As a result, participants who self-reported vaccination but could not provide documented proof were not enrolled. This conservative approach likely led to an underestimation of vaccination coverage in our cohort compared with the national estimate. Among vaccinated individuals, 37.1% received full immunisation according to the schedule (at least 3 doses). Despite vaccination, only 83.4% of individuals demonstrated detectable TBEV-specific IgG antibodies by the standard ELISA, which is likely attributable to incomplete vaccination schedules, waning immunity without timely boosters, or individual variability in immune responses. A small proportion of vaccinated individuals (7.0%) were NS1-positive, which indicates that these individuals were likely exposed to natural TBEV infection despite having been vaccinated. However, the NS1 IgG ELISA test does not indicate the exact timing of exposure. This could reflect breakthrough infections or exposures occurring before full vaccine-induced immunity developed. The ability of the NS1 ELISA to detect such cases highlights its utility in identifying natural infections that would otherwise be indistinguishable in vaccinated individuals using conventional serological methods. Furthermore, the results show that the detectable antibodies for NS1 were significantly higher in vaccinated (7.0%) than in unvaccinated individuals (2.9%, *p* < 0.01). This could be explained by prior natural infections preceding vaccination or high exposure risk despite vaccination, rather than cross-reactivity.

The Latvian Biobank samples, analysed separately due to unknown vaccination status, further confirmed the high background seroprevalence, with 32.5% positivity by the standard ELISA and 6.0% NS1 positivity. This underscores the robustness of our findings across independent sample sets, although the lack of detailed clinical data and vaccination status from Biobank participants limits deeper interpretation.

TBEV seroprevalence in the population of Latvia is higher than reports from other countries. When compared to other European countries, the 16.3% seroprevalence measured by the standard ELISA among unvaccinated individuals in Latvia appears notably elevated compared to the meta-analysis led by Kelly et al., evaluating TBEV seroprevalence in Europe and other countries [16]. In Western and Central Europe, the aggregated anti-TBEV seroprevalence across the region was 2.7% (95% CI: 2.1–3.3%; I2 = 94%) for general populations and 4.6% (95% CI: 3.4–6%; I2 = 97%) for high-risk populations. However, there are particular historical reports with the highest reported anti-TBEV seroprevalence rate of 33.4% in high-risk groups (farmers and foresters) from Lower Franconia, Germany [17]. In Scandinavia, aggregated seroprevalence across the region was 1.9% (95% CI: 0.9–3.2%; I2 = 93%) for general populations and 3.4% (95% CI: 1.1–6.7%; I2 = 95%) for high-risk populations. However, a study in Sweden (Albinsson et al.) reported seroprevalence among all blood donors of 27.5% and 2.4% (1–7%) detectable antibodies against the TBEV NS1 antigen measured by TBEV suspension multiplex immunoassay [18]. In Poland, TBEV seroprevalence among unvaccinated blood donors was 5%, and 2% among children, suggesting a history of undiagnosed TBEV infection [19]. In North Western Romania, only 0.1% (1/1200) of blood donors were seropositive [20]. This gives us insight into the fact that TBEV seroprevalence is not uniform across Europe, even between relatively close geographic areas, and highlights the importance of tailored public health strategies to manage TBEV risk within a country and collectively.

Subtype-specific analysis by the NS1 IgG ELISA indicated the presence of all three TBEV subtypes (EU, Sib, FE) among Latvian residents, consistent with historical reports. However, further data are needed to clarify the single Far Eastern subtype case to determine whether it reflects a true infection or is the result of cross-reactivity or other limitations of the assay. Although subtype differentiation was not the primary focus, the identification of multiple subtypes has important clinical implications, as infection with different subtypes may lead to varying disease severity and outcomes. TBEV-EU is the predominant subtype in Europe and is generally linked to milder disease manifestations and lower fatality rates (0.5–2%), compared to higher mortality associated with TBEV-Sib and TBEV-FE infections (5–35%) [21,22,23]. In this study, TBEV-EU was the dominant subtype in Latvia (Group 1), accounting for 81.8% (36/44) of residents with NS1 antibodies indicative of past natural infection. TBEV-Sib infections were also identified in 15.9% (7/44) of individuals, occurring at lower but notable frequencies across the country, ranging from 9.1% to 33.3% across regions. The detection of TBEV-Sib and TBEV-FE across Latvia may help explain severe or atypical clinical presentations—such as monophasic illness or pronounced neurological sequelae—observed in some acute TBE cases [4,24]. While management of TBE remains supportive across all subtypes, knowledge of the circulating subtype distribution is valuable for clinicians, as it may guide prognosis, inform patient monitoring strategies, and contribute to public health risk assessment.

Furthermore, our findings reinforce the effectiveness of vaccination programmes in reducing natural TBEV infections, with only a small fraction (4.3%) demonstrating NS1-specific antibodies indicative of true infection. Importantly, current vaccines provide cross-protection against all three subtypes, but confirming their co-circulation in Latvia reinforces the importance of vaccination programmes and strengthens public confidence in vaccine effectiveness. Continued and expanded vaccination coverage is therefore essential to sustain this protective effect.

Several limitations warrant consideration. The NS1 IgG ELISA, although highly promising, remains a relatively new diagnostic tool, and its performance characteristics in diverse field settings require further validation. Sensitivity and antibody persistence following infection are not yet fully characterised. A pilot study reported detectable NS1 antibodies up to 28 years post-infection, with high levels still present at 5 years, borderline levels at 10 years, and varying but detectable levels at 23 and 28 years [9]. However, long-term immune response still remains unclear, and further research to determine the typical duration of NS1 antibody persistence is needed. Another limitation is a reliance on participant-reported vaccination history that introduces potential recall bias. Selection bias in Group 2 (Biobank samples), with unknown vaccination histories, may have influenced comparisons. In Group 1, the participant distribution was designed to reflect Latvia’s demographic structure; the quota-based recruitment approach may have introduced selection bias, as enrolment occurred through healthcare settings rather than through population sampling. While the study was sufficiently powered to detect major differences between age groups and regions, smaller subgroup comparisons should be interpreted with caution. Despite these limitations, this study provides the most detailed population-level assessment of TBEV immunity in Latvia to date.

In conclusion, this study demonstrates a high burden of TBEV exposure in Latvia and highlights a substantial proportion of unvaccinated individuals with serological evidence of infection. These findings reinforce the importance of strengthening TBE vaccination programmes, improving public awareness, and establishing vaccination registries. Furthermore, the use of NS1-based serological assays offers a valuable tool for refining epidemiological surveillance and true infection rates in endemic settings. In clinical practice, NS1 testing may aid in distinguishing between vaccine-induced and natural immunity, supporting more accurate diagnosis and informed patient management. Additionally, the detection of all three TBEV subtypes—European, Siberian, and Far Eastern—underscores the need to consider subtype diversity in both clinical evaluation and public health planning, given the differences in disease severity and outcomes associated with each subtype.

## Figures and Tables

**Table 1:** Questionnaire data on gender, age, and occupational and leisure risk factors of residents in different regions of Latvia.

Residents of Latvia	Total	TBE Vaccination Status *	
		Vaccinated	Non-Vaccinated
	N	*n* (%)	*n* (%)
All	1020	356 (34.9%)	663 (65.0%)
Sex			
Male	412	133 (32.3%)	279 (67.7%)
Female	608	223 (36.7%)	384 (63.1%)
Children (1–17 years old)	186	72 (38.7%)	114 (61.3%)
Adults (18+ years old)	834	284 (34.1%)	549 (65.8%)
18–39	270	102 (37.8%)	168 (62.2%)
40–64	350	126 (36.0%)	223 (63.7%)
65+	214	56 (26.2%)	158 (73.8%)
Region of residence			
Riga	202	64 (31.7%)	137 (67.8%)
Vidzeme	203	118 (58.1%)	85 (41.9%)
Kurzeme	203	72 (35.5%)	131 (64.5%)
Latgale	207	18 (8.7%)	189 (91.3%)
Zemgale	205	84 (41.0%)	121 (59.0%)
Underlying prior conditions	223	78 (35.0%)	145 (65.0%)
HIV	1	0	1
Immunosuppressive medication	12	6	6
Cancer	27	9	18
Other	191	67	124
Occupational risk factors	150	66 (44.0%)	84 (56.0%)
Forest/environmental worker	50	20	30
Hunter	20	7	13
National Armed Forces Personnel	2	2	0
Farmer	86	42	44
Fireman	1	0	1
Veterinarian	1	0	1
Leisure activity risk factors	832	296 (35.6%)	535 (64.3%)
Outdoor sports	295	133	162
Berry/flower/mushroom picking	583	222	360
Walking/hiking	724	269	455
Camping	298	152	146
Fishing	4	1	3
Gardening	38	9	29
Tick bite noticed	425	165 (38.8%)	260 (61.2%)
TBE infection in the past	5	0	5 (100%)

* One patient located in Riga region with unknown vaccination status was not included in the analysis.

**Table 2:** Anti-TBEV IgG antibody prevalence among non-vaccinated residents by standard ELISA and NS1 IgG ELISA.

	Non-Vaccinated Residents, N	Standard ELISA Seroprevalence, *n* (%)	*p*-Value	NS1 IgG ELISA Seroprevalence, *n* (%)	*p*-Value
All	663	108 (16.3%)		19 (2.9%)	
Sex			0.246		1.0
Male	279	40 (14.3%)		8 (2.9%)	
Female	384	68 (17.7%)		11 (2.9%)	
Children	114	18 (15.8%)		0	
Adults	549	90 (16.4%)		19 (3.5%)	
18–39 y.o.	168	37 (22.0%)	0.005	4 (2.4%)	0.515
40–64 y.o.	223	38 (17.0%)		10 (4.5%)	
65+ y.o.	158	14 (8.9%)		5 (3.2%)	
Regions			0.004		0.066
Riga	137	27 (19.7%)		4 (2.9%)	
Vidzeme	85	14 (16.5%)		2 (2.4%)	
Kurzeme	131	17 (13.0%)		8 (6.1%)	
Latgale	189	19 (10.1%)		1 (0.5%)	
Zemgale	121	31 (25.6%)		4 (3.3%)	

## Data Availability

All relevant data are within the manuscript. Primary data cannot be shared publicly because of ethical or legal restrictions. RSU Ethics Committee imposes restrictions on the transfer of primary data outside EU/EEA countries, publishing these data online will automatically violate this policy. Also, Patient Informed Consent includes statement that patient data will only be stored and available for restricted research group. For data availability for researchers who meet the criteria for access to confidential data, please contact Riga Stradins University/Ethics Committee. Link to a website RSU Ethics Committee with contact details for the data requests https://www.rsu.lv/en/research-ethics-committee (accessed on 15 October 2025).
